# Phen­yl(2,4,5-triphenyl­cyclo­penta-1,4-dien-1-yl)methanone

**DOI:** 10.1107/S1600536811022902

**Published:** 2011-06-18

**Authors:** David B. Cordes, Guoxiong Hua, Alexandra M. Z. Slawin, J. Derek Woollins

**Affiliations:** a School of Chemistry, University of St Andrews, Fife KY16 9ST, Scotland

## Abstract

The title compound, C_30_H_22_O, does not form face-to-face π–π inter­actions despite the presence of four phenyl rings within the compound. Instead weak C—H⋯π inter­actions occur between adjacent mol­ecules, with C⋯C contact distances in the range 3.633 (4)–3.974 (4) Å. The ketone O atom also takes part in a weak C—H⋯O inter­action. The three pendant phenyl rings are twisted relative to the central cyclopentadiene ring by 17.82 (17), 29.63 (14) and 61.57 (9)°, while the phenylmethanone is nearly orthogonal, the angle between planes being 87.77 (9)°.

## Related literature

For a previous preparation of the title compound, see: Lund (2005 ▶). The crystal studied was obtained by reaction of Woollins’ reagent [2,4-bis­(phen­yl)-1,3-diselenadiphosphetane-2,4-disel­en­ide] with quinoxaline-2,3-dithiol. For a review of the chemistry of Woollins’ reagent, see: Hua & Woollins (2009 ▶). There are no structurally closely-related compounds in the literature; however, for some of the closest related, see: Evrard *et al.* (1971 ▶); Wender *et al.* (2006 ▶).
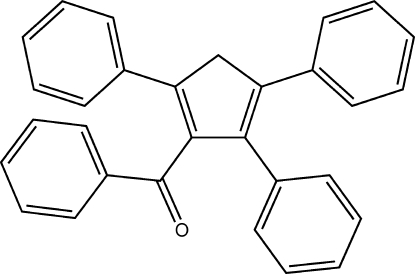


## Experimental

### 

#### Crystal data

C_30_H_22_O
*M* *_r_* = 398.48
Monoclinic, 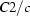

*a* = 25.946 (6) Å
*b* = 6.1573 (14) Å
*c* = 26.602 (6) Å
β = 102.236 (7)°
*V* = 4153.3 (16) Å^3^
*Z* = 8
Mo *K*α radiation
μ = 0.08 mm^−1^
*T* = 93 K
0.30 × 0.20 × 0.06 mm

#### Data collection

Rigaku Mercury CCD diffractometer
Absorption correction: multi-scan (*CrystalClear*; Rigaku, 2010 ▶) *T*_min_ = 0.978, *T*_max_ = 0.996
13266 measured reflections
4252 independent reflections
2479 reflections with *I* > 2σ(*I*)
*R*_int_ = 0.120

#### Refinement

*R*[*F* ^2^ > 2σ(*F* ^2^)] = 0.081
*wR*(*F* ^2^) = 0.225
*S* = 1.04
4252 reflections
281 parameters
H-atom parameters constrained
Δρ_max_ = 0.29 e Å^−3^
Δρ_min_ = −0.39 e Å^−3^

### 

Data collection: *CrystalClear* (Rigaku, 2010 ▶); cell refinement: *CrystalClear*; data reduction: *CrystalClear*; program(s) used to solve structure: *SHELXTL* (Sheldrick, 2008 ▶); program(s) used to refine structure: *SHELXTL*; molecular graphics: *SHELXTL*; software used to prepare material for publication: *SHELXTL*.

## Supplementary Material

Crystal structure: contains datablock(s) global, I. DOI: 10.1107/S1600536811022902/fj2426sup1.cif

Structure factors: contains datablock(s) I. DOI: 10.1107/S1600536811022902/fj2426Isup2.hkl

Supplementary material file. DOI: 10.1107/S1600536811022902/fj2426Isup3.cml

Additional supplementary materials:  crystallographic information; 3D view; checkCIF report

## Figures and Tables

**Table 1:** Hydrogen-bond geometry (Å, °) *Cg*1 and *Cg*2 are the centroids of the C6–C11 and C25–C30 rings, respectively.

*D*—H⋯*A*	*D*—H	H⋯*A*	*D*⋯*A*	*D*—H⋯*A*
C1—H1*B*⋯O1^i^	0.99	2.64	3.229 (3)	118 (2)
C10—H10⋯*Cg*1^ii^	0.95	2.80	3.527 (3)	134 (2)
C20—H20⋯*Cg*2^iii^	0.95	2.80	3.605 (3)	143 (2)
C28—H28⋯*Cg*1^iv^	0.95	2.88	3.612 (3)	134 (2)

Symmetry codes: (i) 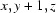; (ii) 
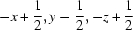
; (iii) 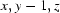; (iv) 
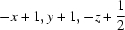
.

**Fig. 1.**
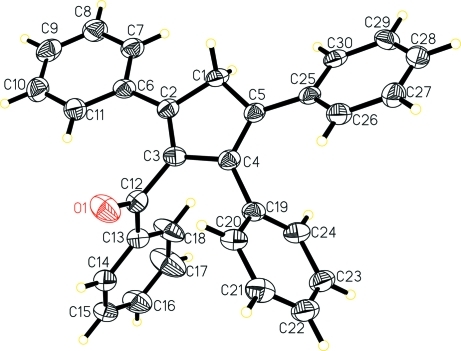
The structure of the title compound with displacement ellipsoids drawn at the 50% probability level.

## supplementary crystallographic information

### Comment

The previously know title compound (Lund, 2005) has been prepared by the reaction of Woollins' reagent with quinoxaline-2,3-dithiol. In a similar manner to the two somewhat related structures (Evrard *et al.*, 1971 and Wender *et al.*, 2006) no face-to-face π-interactions are observed, adjacent molecules instead interacting *via* a series of CH···π interactions. The ketone oxygen makes intermolecular CH···O contacts at a distance of 2.64 Å.

### Experimental

A mixture of 0.194 g of quinoxaline-2,3-dithiol (1.0 mmol) and Woollins' reagent (0.54 g, 1.0 mmol) in 20 ml of dry toluene was refluxed for 7 h. The red suspension disappeared and a deep red solution formed. Following cooling to room temperature and removal of solvent *in vacuuo* the residue was purified by silica gel column chromatography (1: 1 hexane/dichloromethane eluent) to give the title compound as a brown solid (0.060 g, 13%). Crystals suitable for X-ray structure determination were obtained from the diffusion of hexane into a dichloromethane solution of the title compound. Selected IR (KBr, cm^-1^): 1658(s, C═O), 1596(*m*), 1490(*m*), 1443(*m*), 1243(*s*), 754(*s*), 6932(*versus*). ^1^H NMR (CD_2_Cl_2_, δ), 8.13–8.00 (m, 2H, ArH), 7.93–7.83 (m, 3H, ArH), 7.61–6.92 (m, 15H, ArH), 4.24 (s, 2H, CH_2_) p.p.m.. ^13^C NMR (CD_2_Cl_2_, δ), 168.5 (C═O), 144.0, 135.8, 134.4, 133.4, 132.6, 130.7, 129.8, 129.4, 129.2, 129.0, 128.9, 128.8, 128.5, 128.3, 128.1, 127.9, 127.6, 127.4, 127.2, 127.0, 126.6, 46.0 p.p.m.. MS (CI^+^, m/*z*), 399 [*M*+H]^+^. Accurate mass measurement [CI^+^, m/*z*]: 399.1737 [*M*+H]^+^, calculated mass for C_30_H_23_O: 399.1743.

### Refinement

All the crystals chosen appeared to be poorly diffracting at higher angles, with low values of I/σ(I), and missing independent data in the experimentally measured range. All H atoms were included in calculated positions (C—H distances are 0.99 Å for methylene H atoms and 0.95 Å for phenyl H atoms) and refined as riding atoms with *U*_iso_(H) = 1.2 *U*_eq_(parent atom).

### Crystal data

**Table:**

C_30_H_22_O	*F*(000) = 1680
*M**_r_* = 398.48	*D*_x_ = 1.275 Mg m^−^^3^
Monoclinic, *C*2/*c*	Mo *K*α radiation, λ = 0.71073 Å
Hall symbol: -C 2yc	Cell parameters from 3958 reflections
*a* = 25.946 (6) Å	θ = 6.3–54.9°
*b* = 6.1573 (14) Å	µ = 0.08 mm^−^^1^
*c* = 26.602 (6) Å	*T* = 93 K
β = 102.236 (7)°	Prism, colourless
*V* = 4153.3 (16) Å^3^	0.30 × 0.20 × 0.06 mm
*Z* = 8	

### Data collection

**Table:**

Rigaku Mercury CCD diffractometer	4252 independent reflections
Radiation source: rotating anode	2479 reflections with *I* > 2σ(*I*)
confocal	*R*_int_ = 0.120
Detector resolution: 14.7059 pixels mm^-1^	θ_max_ = 27.5°, θ_min_ = 3.1°
ω and φ scans	*h* = −33→26
Absorption correction: multi-scan (*CrystalClear*; Rigaku, 2010)	*k* = −7→7
*T*_min_ = 0.978, *T*_max_ = 0.996	*l* = −28→33
13266 measured reflections	

### Refinement

**Table:**

Refinement on *F*^2^	Primary atom site location: structure-invariant direct methods
Least-squares matrix: full	Secondary atom site location: difference Fourier map
*R*[*F*^2^ > 2σ(*F*^2^)] = 0.081	Hydrogen site location: inferred from neighbouring sites
*wR*(*F*^2^) = 0.225	H-atom parameters constrained
*S* = 1.04	*w* = 1/[σ^2^(*F*_o_^2^) + (0.1072*P*)^2^] where *P* = (*F*_o_^2^ + 2*F*_c_^2^)/3
4252 reflections	(Δ/σ)_max_ < 0.001
281 parameters	Δρ_max_ = 0.29 e Å^−^^3^
0 restraints	Δρ_min_ = −0.39 e Å^−^^3^

### Special details

**Table:**

Geometry. All e.s.d.'s (except the e.s.d. in the dihedral angle between two l.s. planes) are estimated using the full covariance matrix. The cell e.s.d.'s are taken into account individually in the estimation of e.s.d.'s in distances, angles and torsion angles; correlations between e.s.d.'s in cell parameters are only used when they are defined by crystal symmetry. An approximate (isotropic) treatment of cell e.s.d.'s is used for estimating e.s.d.'s involving l.s. planes.
Refinement. Refinement of *F*^2^ against ALL reflections. The weighted *R*-factor *wR* and goodness of fit *S* are based on *F*^2^, conventional *R*-factors *R* are based on *F*, with *F* set to zero for negative *F*^2^. The threshold expression of *F*^2^ > σ(*F*^2^) is used only for calculating *R*-factors(gt) *etc*. and is not relevant to the choice of reflections for refinement. *R*-factors based on *F*^2^ are statistically about twice as large as those based on *F*, and *R*- factors based on ALL data will be even larger.

### Fractional atomic coordinates and isotropic or equivalent isotropic displacement parameters (Å^2^)

**Table:**

	*x*	*y*	*z*	*U*_iso_*/*U*_eq_	
O1	0.36026 (8)	0.0838 (4)	0.13591 (8)	0.0494 (6)	
C1	0.41982 (10)	0.7166 (5)	0.21370 (9)	0.0300 (7)	
H1A	0.4480	0.6866	0.2443	0.036*	
H1B	0.4032	0.8577	0.2185	0.036*	
C2	0.37945 (10)	0.5375 (4)	0.20539 (9)	0.0279 (7)	
C3	0.37812 (10)	0.4503 (4)	0.15812 (9)	0.0289 (7)	
C4	0.41591 (10)	0.5655 (4)	0.13312 (9)	0.0283 (7)	
C5	0.44176 (10)	0.7189 (4)	0.16562 (9)	0.0269 (6)	
C6	0.34825 (10)	0.4843 (5)	0.24389 (10)	0.0304 (7)	
C7	0.34567 (11)	0.6341 (5)	0.28290 (10)	0.0368 (7)	
H7	0.3645	0.7672	0.2842	0.044*	
C8	0.31633 (12)	0.5923 (6)	0.31959 (11)	0.0454 (8)	
H8	0.3153	0.6964	0.3457	0.054*	
C9	0.28875 (12)	0.4015 (6)	0.31855 (11)	0.0452 (8)	
H9	0.2681	0.3746	0.3434	0.054*	
C10	0.29138 (11)	0.2480 (5)	0.28080 (12)	0.0434 (8)	
H10	0.2730	0.1144	0.2803	0.052*	
C11	0.32083 (10)	0.2887 (5)	0.24366 (11)	0.0350 (7)	
H11	0.3223	0.1828	0.2180	0.042*	
C12	0.34422 (11)	0.2708 (5)	0.13193 (10)	0.0315 (7)	
C13	0.29162 (11)	0.3222 (5)	0.09964 (10)	0.0313 (7)	
C14	0.26130 (11)	0.1535 (5)	0.07379 (10)	0.0374 (7)	
H14	0.2746	0.0093	0.0765	0.045*	
C15	0.21138 (11)	0.1970 (5)	0.04388 (11)	0.0416 (8)	
H15	0.1906	0.0819	0.0265	0.050*	
C16	0.19237 (12)	0.4045 (6)	0.03957 (12)	0.0516 (9)	
H16	0.1584	0.4337	0.0191	0.062*	
C17	0.22223 (14)	0.5704 (6)	0.06479 (16)	0.0740 (13)	
H17	0.2090	0.7148	0.0615	0.089*	
C18	0.27205 (12)	0.5288 (5)	0.09539 (12)	0.0522 (10)	
H18	0.2923	0.6443	0.1132	0.063*	
C19	0.42011 (10)	0.5280 (5)	0.07848 (9)	0.0291 (7)	
C20	0.43588 (10)	0.3314 (5)	0.06182 (10)	0.0341 (7)	
H20	0.4437	0.2132	0.0852	0.041*	
C21	0.44040 (11)	0.3058 (5)	0.01114 (11)	0.0397 (8)	
H21	0.4518	0.1709	0.0000	0.048*	
C22	0.42837 (11)	0.4765 (5)	−0.02322 (11)	0.0398 (8)	
H22	0.4315	0.4583	−0.0579	0.048*	
C23	0.41199 (11)	0.6712 (5)	−0.00742 (10)	0.0380 (8)	
H23	0.4035	0.7875	−0.0312	0.046*	
C24	0.40768 (11)	0.6993 (5)	0.04363 (10)	0.0358 (7)	
H24	0.3963	0.8346	0.0546	0.043*	
C25	0.48404 (10)	0.8684 (4)	0.15913 (9)	0.0271 (7)	
C26	0.52110 (10)	0.8136 (5)	0.12983 (10)	0.0336 (7)	
H26	0.5184	0.6775	0.1127	0.040*	
C27	0.56144 (11)	0.9533 (5)	0.12544 (10)	0.0381 (8)	
H27	0.5859	0.9127	0.1051	0.046*	
C28	0.56674 (11)	1.1516 (5)	0.15023 (11)	0.0392 (8)	
H28	0.5946	1.2473	0.1471	0.047*	
C29	0.53077 (11)	1.2092 (5)	0.17984 (11)	0.0373 (8)	
H29	0.5341	1.3447	0.1973	0.045*	
C30	0.49004 (11)	1.0692 (5)	0.18395 (9)	0.0317 (7)	
H30	0.4656	1.1110	0.2042	0.038*	

### Atomic displacement parameters (Å^2^)

**Table:**

	*U*^11^	*U*^22^	*U*^33^	*U*^12^	*U*^13^	*U*^23^
O1	0.0409 (12)	0.0385 (14)	0.0614 (14)	0.0034 (10)	−0.0057 (11)	−0.0054 (11)
C1	0.0278 (15)	0.0406 (18)	0.0201 (14)	0.0017 (12)	0.0013 (11)	0.0012 (11)
C2	0.0215 (14)	0.0374 (18)	0.0244 (15)	0.0014 (11)	0.0041 (11)	0.0016 (11)
C3	0.0221 (14)	0.0344 (17)	0.0271 (15)	0.0022 (11)	−0.0017 (11)	0.0006 (12)
C4	0.0243 (14)	0.0341 (17)	0.0251 (14)	0.0014 (11)	0.0019 (11)	0.0019 (12)
C5	0.0227 (14)	0.0343 (17)	0.0223 (14)	0.0008 (11)	0.0016 (11)	0.0032 (11)
C6	0.0197 (14)	0.0410 (18)	0.0280 (15)	0.0001 (12)	−0.0003 (11)	0.0011 (12)
C7	0.0349 (17)	0.048 (2)	0.0280 (15)	−0.0041 (14)	0.0078 (13)	−0.0019 (13)
C8	0.047 (2)	0.057 (2)	0.0338 (17)	−0.0068 (16)	0.0134 (14)	−0.0049 (15)
C9	0.0383 (18)	0.068 (2)	0.0318 (17)	−0.0027 (16)	0.0139 (14)	0.0047 (16)
C10	0.0355 (18)	0.053 (2)	0.0419 (18)	−0.0084 (15)	0.0078 (14)	0.0089 (16)
C11	0.0307 (16)	0.0411 (19)	0.0337 (16)	−0.0035 (13)	0.0079 (13)	−0.0009 (13)
C12	0.0314 (16)	0.0336 (18)	0.0300 (15)	0.0018 (13)	0.0079 (12)	0.0020 (12)
C13	0.0323 (16)	0.0339 (17)	0.0270 (15)	0.0001 (12)	0.0044 (12)	−0.0020 (12)
C14	0.0387 (17)	0.0398 (19)	0.0317 (16)	−0.0051 (14)	0.0031 (13)	−0.0001 (13)
C15	0.0395 (18)	0.048 (2)	0.0330 (17)	−0.0103 (15)	−0.0032 (13)	−0.0044 (14)
C16	0.0402 (19)	0.054 (2)	0.050 (2)	0.0045 (16)	−0.0141 (15)	−0.0040 (17)
C17	0.055 (2)	0.047 (2)	0.097 (3)	0.0130 (18)	−0.034 (2)	−0.014 (2)
C18	0.0411 (19)	0.040 (2)	0.063 (2)	0.0018 (15)	−0.0173 (16)	−0.0145 (16)
C19	0.0254 (14)	0.0352 (18)	0.0242 (14)	−0.0018 (12)	−0.0003 (11)	−0.0022 (12)
C20	0.0331 (16)	0.0387 (19)	0.0277 (16)	0.0003 (13)	0.0001 (12)	−0.0043 (12)
C21	0.0349 (17)	0.045 (2)	0.0384 (18)	0.0007 (14)	0.0064 (14)	−0.0093 (14)
C22	0.0372 (17)	0.054 (2)	0.0269 (15)	−0.0033 (15)	0.0041 (13)	−0.0038 (14)
C23	0.0385 (17)	0.049 (2)	0.0253 (16)	−0.0016 (14)	0.0041 (13)	0.0006 (13)
C24	0.0344 (16)	0.0428 (19)	0.0273 (16)	0.0032 (13)	0.0000 (12)	−0.0018 (13)
C25	0.0233 (14)	0.0365 (17)	0.0191 (14)	0.0002 (12)	−0.0010 (11)	0.0034 (11)
C26	0.0308 (16)	0.0416 (19)	0.0273 (15)	−0.0013 (13)	0.0039 (12)	−0.0029 (12)
C27	0.0331 (17)	0.050 (2)	0.0319 (16)	−0.0055 (14)	0.0086 (13)	0.0022 (14)
C28	0.0299 (17)	0.051 (2)	0.0323 (17)	−0.0089 (14)	−0.0022 (13)	0.0075 (14)
C29	0.0334 (17)	0.0406 (19)	0.0330 (16)	−0.0055 (13)	−0.0046 (13)	−0.0018 (13)
C30	0.0335 (16)	0.0411 (19)	0.0178 (13)	0.0022 (13)	−0.0005 (11)	0.0025 (12)

### Geometric parameters (Å, °)

**Table:**

O1—C12	1.221 (3)	C15—H15	0.9500
C1—C2	1.504 (4)	C16—C17	1.369 (5)
C1—C5	1.506 (4)	C16—H16	0.9500
C1—H1A	0.9900	C17—C18	1.398 (4)
C1—H1B	0.9900	C17—H17	0.9500
C2—C3	1.361 (4)	C18—H18	0.9500
C2—C6	1.471 (4)	C19—C20	1.381 (4)
C3—C4	1.478 (4)	C19—C24	1.396 (4)
C3—C12	1.490 (4)	C20—C21	1.387 (4)
C4—C5	1.358 (3)	C20—H20	0.9500
C4—C19	1.498 (4)	C21—C22	1.384 (4)
C5—C25	1.470 (4)	C21—H21	0.9500
C6—C11	1.398 (4)	C22—C23	1.367 (4)
C6—C7	1.401 (4)	C22—H22	0.9500
C7—C8	1.383 (4)	C23—C24	1.397 (4)
C7—H7	0.9500	C23—H23	0.9500
C8—C9	1.372 (4)	C24—H24	0.9500
C8—H8	0.9500	C25—C30	1.395 (4)
C9—C10	1.392 (4)	C25—C26	1.401 (4)
C9—H9	0.9500	C26—C27	1.379 (4)
C10—C11	1.394 (4)	C26—H26	0.9500
C10—H10	0.9500	C27—C28	1.380 (4)
C11—H11	0.9500	C27—H27	0.9500
C12—C13	1.484 (4)	C28—C29	1.389 (4)
C13—C18	1.366 (4)	C28—H28	0.9500
C13—C14	1.393 (4)	C29—C30	1.386 (4)
C14—C15	1.395 (4)	C29—H29	0.9500
C14—H14	0.9500	C30—H30	0.9500
C15—C16	1.366 (4)		
			
C2—C1—C5	105.0 (2)	C14—C15—H15	119.9
C2—C1—H1A	110.7	C15—C16—C17	120.0 (3)
C5—C1—H1A	110.7	C15—C16—H16	120.0
C2—C1—H1B	110.7	C17—C16—H16	120.0
C5—C1—H1B	110.7	C16—C17—C18	120.4 (3)
H1A—C1—H1B	108.8	C16—C17—H17	119.8
C3—C2—C6	130.2 (3)	C18—C17—H17	119.8
C3—C2—C1	107.8 (2)	C13—C18—C17	120.1 (3)
C6—C2—C1	122.0 (2)	C13—C18—H18	120.0
C2—C3—C4	109.7 (2)	C17—C18—H18	120.0
C2—C3—C12	128.4 (3)	C20—C19—C24	119.5 (3)
C4—C3—C12	121.9 (2)	C20—C19—C4	122.2 (2)
C5—C4—C3	109.4 (2)	C24—C19—C4	118.3 (2)
C5—C4—C19	126.7 (2)	C19—C20—C21	120.2 (3)
C3—C4—C19	123.7 (2)	C19—C20—H20	119.9
C4—C5—C25	129.9 (2)	C21—C20—H20	119.9
C4—C5—C1	108.0 (2)	C22—C21—C20	120.1 (3)
C25—C5—C1	122.1 (2)	C22—C21—H21	119.9
C11—C6—C7	117.8 (3)	C20—C21—H21	119.9
C11—C6—C2	122.9 (3)	C23—C22—C21	120.3 (3)
C7—C6—C2	119.2 (3)	C23—C22—H22	119.9
C8—C7—C6	121.3 (3)	C21—C22—H22	119.9
C8—C7—H7	119.3	C22—C23—C24	120.1 (3)
C6—C7—H7	119.3	C22—C23—H23	120.0
C9—C8—C7	120.5 (3)	C24—C23—H23	120.0
C9—C8—H8	119.7	C19—C24—C23	119.8 (3)
C7—C8—H8	119.7	C19—C24—H24	120.1
C8—C9—C10	119.4 (3)	C23—C24—H24	120.1
C8—C9—H9	120.3	C30—C25—C26	117.1 (2)
C10—C9—H9	120.3	C30—C25—C5	120.7 (2)
C9—C10—C11	120.5 (3)	C26—C25—C5	122.1 (2)
C9—C10—H10	119.8	C27—C26—C25	121.3 (3)
C11—C10—H10	119.8	C27—C26—H26	119.4
C10—C11—C6	120.5 (3)	C25—C26—H26	119.4
C10—C11—H11	119.8	C26—C27—C28	120.8 (3)
C6—C11—H11	119.8	C26—C27—H27	119.6
O1—C12—C13	120.4 (3)	C28—C27—H27	119.6
O1—C12—C3	120.1 (2)	C27—C28—C29	119.1 (3)
C13—C12—C3	119.4 (2)	C27—C28—H28	120.5
C18—C13—C14	119.5 (3)	C29—C28—H28	120.5
C18—C13—C12	121.8 (2)	C30—C29—C28	120.1 (3)
C14—C13—C12	118.7 (3)	C30—C29—H29	120.0
C13—C14—C15	119.8 (3)	C28—C29—H29	120.0
C13—C14—H14	120.1	C29—C30—C25	121.6 (3)
C15—C14—H14	120.1	C29—C30—H30	119.2
C16—C15—C14	120.2 (3)	C25—C30—H30	119.2
C16—C15—H15	119.9		

### Hydrogen-bond geometry (Å, °)

**Table:**

Cg1 and Cg2 are the centroids of the C6–C11 and C25–C30 rings, respectively.

**Table:**

*D*—H···*A*	*D*—H	H···*A*	*D*···*A*	*D*—H···*A*
C1—H1B···O1^i^	0.99	2.64	3.229 (3)	118.(2)
C10—H10···Cg1^ii^	0.95	2.80	3.527 (3)	134 (2)
C20—H20···Cg2^iii^	0.95	2.80	3.605 (3)	143 (2)
C28—H28···Cg1^iv^	0.95	2.88	3.612 (3)	134 (2)
C10—H10···C10^ii^	0.95	3.06	3.908 (4)	150 (2)
C20—H20···C30^iii^	0.95	2.79	3.633 (4)	148 (2)
C28—H28···C9^iv^	0.95	3.12	3.974 (4)	151 (2)

Symmetry codes: (i) *x*, *y*+1, *z*; (ii) −*x*+1/2, *y*−1/2, −*z*+1/2; (iii) *x*, *y*−1, *z*; (iv) −*x*+1, *y*+1, −*z*+1/2.
